# In silico transcriptional regulation and functional analysis of dengue shock syndrome associated SNPs in *PLCE1* and *MICB* genes

**DOI:** 10.1007/s10142-016-0489-9

**Published:** 2016-04-01

**Authors:** Malik Mumtaz Taqi, Durdana Waseem, Humaira Ismatullah, Syed Aleem Haider, Muhammad Faisal

**Affiliations:** Division of Mental Health and Addiction, NORMENT, University of Oslo, Oslo, Norway; Department of Pharmacy, Quaid-i-Azam University, Islamabad, Pakistan; Research Center for Modelling and Simulation (RCMS), National University of Science and Technology, Islamabad, Pakistan; National Center for Bioinformatics, Quaid-i-Azam University, Islamabad, Pakistan; Faculty of Health Studies, University of Bradford, BD7 1DP Bradford, UK; Bradford Institute for Health Research, Bradford Teaching Hospitals NHS Foundation Trust, Bradford, UK

**Keywords:** Phospholipase C epsilon (*PLCE1*), *MICB*, Transcription factors, Dengue-associated SNPs, Mutated PLCε protein

## Abstract

Single nucleotide polymorphisms (SNPs) in *PLCE1* and *MICB* genes increase risk for the development of dengue shock syndrome (DSS). We used Bioinformatics tools to predict alterations at the transcriptional and posttranslational levels driven by *PLCE1* and *MICB* SNPs associated with DSS. Functional and phenotypic analysis conducted to determine deleterious SNPs and impact of amino acid substitution on the structure and function of proteins identified rs2274223 (H1619R) as deleterious to protein coding as it induces structural change in the C2 domain of PLCε, with the mutant residue more positively charged than the wild-type residue (RMSD score, 1.75 Å). Moreover, rs2274223 condenses the chromatin-repressing PLCε expression in DSS. Briefly, this study presents the impact of a single nucleotide transition at SNPs associated with DSS on differential protein binding patterns with *PLCE1* and *MICB* genes and on protein structure modification and their possible role in the pathogenesis of DSS.

## Introduction

Dengue fever is a mosquito-borne acute viral infection that affects infants, young children, and adults (Khor et al. 2011). Since the dawn of the twenty-first century, both dengue fever and dengue shock syndrome (DSS) have emerged as the most important arboviral diseases that have threatened more than 2.5 billion people across the world (Gubler 2002). Dengue fever results in a wide spectrum of clinical manifestations ranging from mild flu-like symptoms to severe DSS which is characterized by coagulopathy and increased vascular permeability (Harris et al. 1998). The prime targets of infection are epidermal dendritic cells, keratinocytes, and lymphatic, splenic, and liver monocytes and macrophages (Blackley et al. 2007; Kou et al. 2008; Limon-Flores et al. 2005). Following infection, abortively infected cells produce bulk of inflammatory cytokines and chemokines that modify host hemostatic response (Huerta-Zepeda et al. 2008). Among other factors, major histocompatibility complex (MHC) and enzymes for the production of inflammatory mediators such as phosphoinositide-specific phospholipase C (PLC) play crucial roles.

Amid the six families of mammalian PLC isoforms (β, γ, δ, ε, ζ, and η), phospholipase C epsilon (PLCε) encoded by the *PLCE1* gene is key downstream effectors of the Ras family proteins (Kelley et al. 2001; Rhee and Bae 1997; Suh et al. 2008). Recent research has reported the role of PLCε in inflammatory reactions, tumor necrosis alpha-induced chemokine expression, and tumorigenesis (Bai et al. 2004; Cheng et al. 2011; Harada et al. 2011). In addition, MHC class I (MHC-I) proteins encoded by the MHC-I chain-related sequence B (*MICB*) gene, present in natural killer (NK) cells and cytotoxic T (CD8^+^) cells (Steinle et al. 2001), are responsible for presenting antigenic proteins of dengue virus to lymphocytes for immune modulation. Susceptibility to dengue-mediated hypovolemic shock, clinical picture, and severity of the disease is influenced by the genetic polymorphism in individuals. Single nucleotide polymorphism (SNP) in *PLCE1* and *MICB* genes has been associated with the development of DSS (Khor et al. 2011).

In a genome-wide association study (GWAS) conducted in Vietnam, 2,118 DSS patients (children) and 2,089 healthy controls were genotyped to reveal genetic polymorphism, SNPs, in *MICB* and *PLCE1* genes. The study has reported a significant association of *MICB* SNP rs3132468 and *PLCE1* SNP rs3740360 with DSS in children. The epidemiology, medical history, inclusion criteria, and virogical and clinical characteristics of the patients and control population who participated in the study can be assessed in the supplementary information (Khor et al. 2011). Another large study using 3,961 dengue fever patients and 5,968 controls has established that these SNPs are significantly associated with non-severe dengue fever in adults, children, and infants (Whitehorn et al. 2013). Apart from these SNPs, 12 *MICB* and 6 *PLCE1* SNPs have also shown significant association with DSS (Khor et al. 2011).

Despite the association of *MICB* and *PLCE1* SNPs with DSS, the underlying molecular mechanisms for the genesis and progression of the disease are not fully understood. Current evidences suggest that SNPs may also alter chromatin remodeling and 3D chromosomal cross talks (Göndör and Ohlsson 2009). It suggests that the transition of single nucleotide at SNPs may enhance or reduce the affinity of transcription factors to regulatory sites (formed by these SNPs), while they may facilitate or inhibit loop and/or bridge formation (3D chromosomal cross talks) among regulatory sites located in different chromosomes, entailing another layer of regulatory mechanisms in modulating gene expression and vulnerability to pathologies, like DSS. Here, we hypothesize that single nucleotide transition at *MICB* and *PLCE1* SNPs significantly associated with DSS may change the pattern of DNA–protein interactions at these SNPs in an allele-specific manner, which in turn alter gene function and, consequently, susceptibility to pathogenesis of DSS. The present study predicts the implication of single nucleotide transition in differential protein binding pattern with *PLCE1* and *MICB* genes and the deleterious effects of these SNPs on the encoded proteins and mutations induced in PLCε structure, leading to changes in protein–ligand interactions. It also determined the effect of our GWAS SNPs on chromatin structure modulating the pathogenesis of DSS.

## Materials and methods

### Sample information

The current study predicts possible mechanisms for the association of *PLCE1* and *MICB* SNPs in the development of DSS. We use data from a GWAS conducted in Vietnam, where 2,118 DSS patients (children) and 2,089 healthy controls were genotyped to reveal genetic polymorphism, SNPs, in *MICB* and *PLCE1* genes. The epidemiology, medical history, inclusion criteria, and virogical and clinical characteristics of the patients and control population who participated in the study can be assessed in the supplementary information (Khor et al. 2011). Briefly, 51 % of the participants are males while 49 % are females. Dengue virus (DNV)-1 induces DSS in 971 patients and 459 DSS patients were infected by DNV-2. Moreover, DNV-3 and DNV-4 trigger DSS in 67 and 60 patients, respectively. Several patients also have mixed infection. Eighty-three percent of the DSS patients have poor peripheral perfusion (Khor et al. 2011).

### SNP datasets

The dataset comprises previously reported *PLCE1* and *MICB* SNPs (Khor et al. 2011) that were significantly associated with DSS. These SNPs were collected from the National Center for Biotechnology Information (NCBI) database of SNPs, dbSNP (http://www.ncbi.nlm.nih.gov/projects/SNP), for our computational analysis. The collected SNPs were (1) intron variants and (2) non-synonymous (ns).

### Identification of functional SNPs in un-translated regions

The potential phenotypic effects of the SNPs present in un-translated regions (UTRs) and in exons were determined by the F-SNP (http://compbio.cs.queensu.ca/F-SNP/) database, which analyzes the integration of 16 bioinformatics tools and predicts functional effects at the splicing, transcriptional, and translational levels. SNP Id was used as query and a functional significance (FS) score ranging between 0 and 1 was retrieved to determine the potential deleterious effects of a SNP in the genomic region. A FS of 0 indicates no deleterious effect predicted by tools, while the higher the score, the greater the predicted deleterious effect of SNP.

SNPeffect 4.0 (http://snpeffect.switchlab.org/), a web-based tool (De Baets et al. 2012), was used to annotate coding SNPs using SNP Ids. The algorithms in SNPeffect are FoldX (determine stability change of protein caused by the single amino acid mutation) and Tango (determine β-aggregation regions in protein sequences), which predict the effect of SNPs according to phenotypic properties like structural dynamics and functional sites.

### Identification of the phenotypic effect of SNPs

The SIFT (Sorting Intolerant From Tolerant) and PolyPhen (Polymorphism Phenotyping) (http://coot.embl.de/PolyPhen/) tools were used to determine the deleterious characteristics of SNPs (Ramensky et al. 2002). We input SNP Ids and selected SWISS-PROT and TrEMBL databases with default settings (3.00 median conservation score, remove sequences >90 % identical to query sequence) for SIFT analysis, while protein sequence was provided as the input to PolyPhen, with mutational position and amino acid variants. SIFT scores are classified as tolerant (0.201–1.00), borderline (0.101–0.20), potentially intolerant (0.051–0.10), or intolerant (0.00–0.05; Ng and Henikoff 2003; Xi et al. 2004). A particular amino acid substitution is likely to have less functional impact if the tolerance index is high and vice versa. The PolyPhen server considers the physiochemical differences, evolutionary conservation, and substitution proximity to the structural features of protein and predicts the effect of amino acid substitution (AAS). PANTHER was used to validate the functional significance of a gene SNP (Mi et al. 2013) based upon hidden Markov model, which predicts output as the substitution position-specific evolutionary conservation (subPSEC) score and probability score of deleterious SNPs. These subPSEC scores are continuous values from −10 (most deleterious) to 0 (neutral). The greater the value of the *P*_deleterious_ score, the higher the tendency of a variant to have severe impairments in protein function (Brunham et al. 2005). Risk associated with AAS was determined by VarioWatch (http://genepipe.ncgm.sinica.edu.tw/variowatch/main.do) by submitting SNP Id with upstream 5′ and downstream 3′ regions as 50,000 bp. VarioWatch analyzes AAS using integrated databases such as dbSNP, Gene Ontology, KEGG, and UniProt (Cheng et al. 2012).

In addition, we used RegulomeDB (http://regulomedb.org), which utilizes CHIP-seq data and chromatin state information across many cell types to determine the effects of our GWAS SNPs on chromatin structure.

### Identification of SNP effect on protein structure

To further illustrate the impact of mutation on the 3D structure and folding of protein, the specific coding regions of PLCε were modeled using homology modeling structure prediction method via the MODELLER tool (Martí-Renom et al. 2000). The sequence of region 1564–1676 (113 amino acids), named “*C2*_*PLC*_*like*” (C2 domain present in PLC), was submitted to Basic Local Alignment Search Tool (BLAST) at the NCBI database. Protein template PDB ID 1DJG (Phosphoinositide-specific Phospholipase C-Delta1 from Rat Complexed with Lanthanum) was selected based on the highest sequence identity and smallest distance on the phylogenetic tree. Structural alignment models were produced by aligning the template and *PLCE1*/“*C2*_*PLC*_*like*” region using align2d command. The MODELLER-calculated energy score and the ERRAT score were used as the criteria for model selection.

The deleterious mutation was mapped and the RMSD between the native and mutated proteins was calculated to investigate changes in the structure, function, and physiochemical properties. The NOMAD-Ref server was used as the energy minimization tool of 3D structures using Gromacs as the default force with conjugate gradient and L-BFGS methods. Differences between the two structures were evaluated by their RMSD values.

Solvent accessibility of the protein was checked by NetASA (neural network-based prediction of solvent accessibility) view, providing the coordinate file of protein as the input (Ahmad and Gromiha 2002). For identification of the stabilizing residues in both native and mutant modeled structures, we used the server SRide with parameters such as stabilization center, surrounding hydrophobicity, long-range order, and conservation score (Magyar et al. 2004, 2005).

The effect of mutation on protein structure stability was also determined using SNPs3D online tool (Yue et al. 2006). This tool uses two models for prediction of SNP effect. The stability model uses a set of factors for approximation of stability effect on the protein structure of a SNP (Peng et al. 2005). In the Stability model, the separation pattern between the diseased and non-deleterious SNPs is identified by SVM (Support Vector Machine). In the Profile model, homology sequence-based analysis is done related to human proteins. On the basis of conservation level at SNP position and variation probability, disease mutations and non-deleterious mutations are distinguished (Peng and John 2006).

### Transcription factor binding sites

To predict the transcription factor binding sites (TFBS) in DNA sequences, Patch 1.0, a pattern-based program, was used. It uses positional weight matrices and a set of binding sites from TRANSFAC® Public 6.0 (Wingender et al. 2000). Query sequence was provided in FASTA format using the following parameters: minimum length of 4, maximum number of mismatches of 2, and mismatch penalty value of 100, with the lower score boundary set to 100. For more precise prediction, we set the lower score boundary to 100, so the estimated score for every match obtained was higher than or equal to this cutoff.

## Results

Genetic polymorphism in the intronic region of *MICB* and *PLCE1* genes increases the risk of DSS by the allele-specific protein–protein and DNA–protein interactions. In the current study, we found that intronic and missense mutations in *MICB* and *PLCE1* genes alter the transcription factor binding patterns, modify histones, and are deleterious to the expressed protein.

### Transcriptional regulation of *MICB* and *PLCE1* genes

**Fig. 1:**
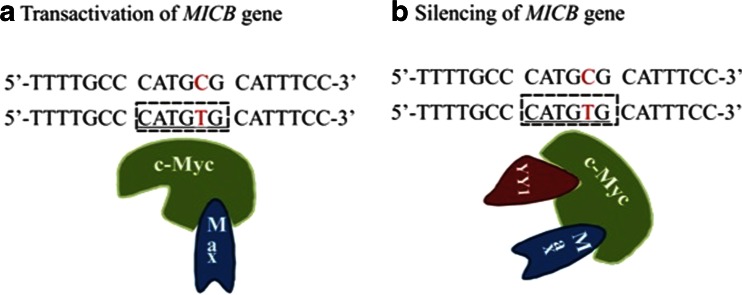
Transcription regulation of *MICB* associated with SNP rs3134899. **a** The c-Myc–MAX complex activates *MICB* gene transcription. **b** Attachment of YY1 with Myc–MAX inactivates this complex, causing silencing of the *MICB* gene. C>T alteration in SNP rs3134899 forms binding motifs for both c-Myc and YY1; therefore, silencing of the *MICB* gene is postulated

**Fig. 2:**
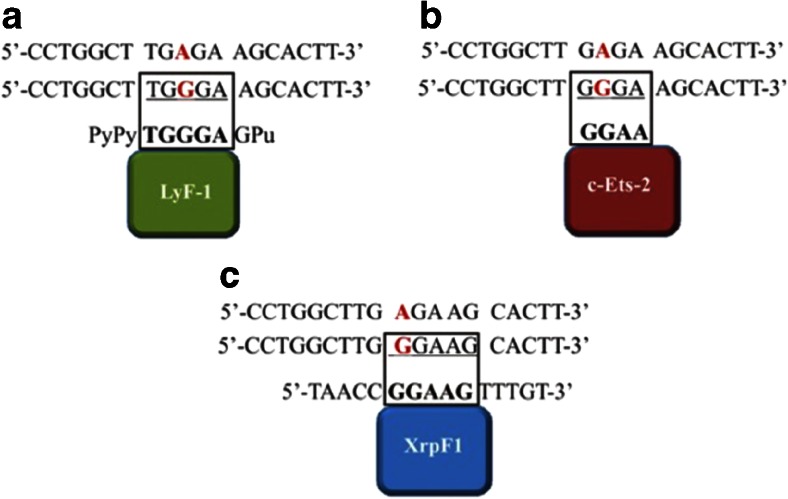
DNA binding motifs for LyF-1, c-Ets-2, and XrpFI formed by *MICB* SNP rs3132468. Alteration from the A>G allele creates canonical and non-canonical binding motifs for c-Ets-2, LyF-1, and XrpFI, respectively. **a** LyF-1 binding to the G-allele of *MICB* SNP rs3132468. **b** c-Ets-2 binding to the G-allele of rs3134899. **c** XrpF1 binding to the G-allele of rs3134899. *Bold* and *underlined* sequences in transcription factors indicate the matching sequence created due to A>G transformation in SNP rs3132468

Although profound knowledge concerning the underlying molecular mechanisms for both dengue fever and DSS is still missing, functional polymorphism in *MICB* and *PLCE1* genes alter the binding affinity of transcription factors in an allele-specific manner and shape susceptibility to DSS (Table 1). The T-allele of rs3134899 forms myelocytomatosis oncogene (Myc) canonical E box (5′-CACGTG-3′; Fig. 1), while the G-allele forms non-canonical consensus sequence for lymphoid transcription factor 1 (LyF-1; motif PyPyTGGGAGPu), E26 transformation-specific factor 2 (c-Ets-2; motif 5′-GGAA\T-3), and *Xenopus laevis* oocyte transcription factors (XrpFI; motif GGAA/T; Fig. 2a–c). These proteins likely underline the molecular mechanism for the development of DSS by regulating expression of the *MICB* gene. Moreover, the presence of the Myc E box in rs3134899 suggests that Myc-Max heterodimer binds to the *MICB* gene and is silenced by YY1 co-repressor (Fig. 1) either directly or through recruiting JunD. This, in turn, reduces *MICB*-stimulated cytokine expression in NK cells in response to dengue virus, resulting in a higher viral burden. This supports the dysfunctional activity of NK and CD8^+^ cells in dengue fever and DSS (Khor et al. 2011; Libraty et al. 2002). Evidences support the notion that SNPs significantly associated with dengue-related pathologies show allele-specific differential affinities for transcription factors (Sakuntabhai et al. 2005).

**Table 1:** Allele-specific interactions of *MICB* and *PLCE1* SNPs with protein factors

Gene	SNP	Ch#: Position (hg19)	Allele	MAF cases	Function	Binding proteins
*MICB*	rs3132468	6: 31475486	A	0.176	Intron variant	–
			G			T3R-alpha, AP-1, CCAAT binding factor, LyF-1, LUN-1, c-Ets-2, HrpF, XrpFI
	rs3134899	6: 31473286	C	0.130	Intron variant	–
			T			YY1, c-Myc, Max
*PLCE1*	rs3765524	10: 96058298	C	0.249	Missense	CAR, RAR-beta, RXR-alpha, LF-A1, RAR-gamma, ROR-alpha1, LXR-alpha, LXR-beta, FXR, PXR-1, c-Fos, c-Jun, AP-1, VDR, TR2-11
			T			GR-alpha, GR-beta, AP-1
	rs2274223	10: 96066341	A	0.250	Missense	–
			G			Sp1, Sp2, Sp3, Sp4
	rs3740360	10: 96025491	A	0.219	Intron variant	ESR-1, ESR-2, LF-A1, Sp1, SRF
			C			ESR-1, ESR-2, LF-A1, Sp1, VDR
	rs12263737	10: 96044913	A	0.250	Intron variant	TFIID, TBP, F2F
			G			TFIID, TBP
	rs11187842	10: 96052511	C	0.219	Intron variant	–
			T			T3R-beta1, ISGF-3, NF-ATc, NF-Atx
	rs753724	10: 96051417	G	0.219	Intron variant	–
			T			SRF
	rs3781264	10: 96070375	C	0.229	Intron variant	Pax-2, Pax-5, Pax-8, SMAD-3, SMAD-4
			T			–

– indicates no bindings

*MAF cases* minor allele frequency in DSS cases

Similarly, it was found that G-to-T transition in *PLCE1* gene SNP rs753724 forms a non-canonical binding motif for SRF. SRF recruits proteins FBJ murine osteosarcoma viral oncogene homolog (Fos) and jun proto-oncogene (Jun; Perez-Albuerne et al. 1993; Treisman 1986) to regulate various molecular pathways. It can also regulate inflammatory processes in severe complications of dengue in a similar manner. Activator protein-1 (AP1) either directly binds with the *PLCE1* gene at the non-canonical binding site formed by the T-allele, and it may regulate gene transcription by interacting with transcription factors—estrogen receptor (ESR) 2, ESR1, and specificity protein (Sp1). Moreover, vitamin D receptor (VDR) may directly interact at rs3740360 and in turn recruit transcription factors to *PLCE1* gene. These VDR-RXR (retinoid X receptor) heterodimers suppress the nuclear factor of activated T cells (NF-AT)-mediated activation of T cells (Hao et al. 2003; Takeuchi et al. 1998). It is proposed that NF-AT, SRF, and VDR may be involved in the regulation of immune response against dengue-mediated hypovolemic shock.

### Functional SNPs in un-translated regions

UTRs or intronic variants were analyzed to determine their potential phenotypic effects due to their importance in translational efficiency and posttranscriptional regulation. Polymorphism in 3′-UTR may regulate transcription efficacy (Barsanjit 2003), while polymorphism in the polyadenylation signal may influence RNA half-life by interfering with polyadenylation. *PLCE1* SNPs were significantly associated (FS score of 0.176) with variation in transcriptional regulation and gene splicing. *MICB* SNPs rs3132468 and rs3134899 were not identified by the F-SNP server (Table 2). This supports the above-mentioned alteration in the binding patterns of transcription factors and indicates that changes in transcriptional regulation of the *PLCE1* gene modify the expression of PLC protein, which is a key signal for the proliferation of CD8^+^ cells. It is documented that PLC is required for the proliferation of T cells in response to infections (Fu et al. 2010). SNP-mediated reduced transcription of the *PLCE1* gene may dampen immune response against dengue virus.

**Table 2:** Functional effect of intronic SNPs predicted by F-SNP

Gene	SNP	Allele	FS score	Prediction tool	Prediction	Functional category
*MICB*	rs3132468	A/G	–	–		Not found in database
	rs3134899	C/T	–	–		Not found in database
*PLCE1*	rs3740360	A/C	0.176	TFSearch	Changed	Transcriptional regulation
	rs12263737	A/G	0.176	TFSearch	Changed	Transcriptional regulation
	rs11187842	C/T	0.176	TFSearch	Changed	Transcriptional regulation
	rs753724	G/T	0.176	TFSearch	Changed	Transcriptional regulation
	rs3781264	C/T	0.176	TFSearch	Changed	Transcriptional regulation

### Deleterious and damaging nsSNP

Missense *PLCE1* SNPs rs3765524 and rs2274223 change the coding protein by shifting gene splicing. Exonic splicing enhancer (ESE) belongs to a conserved splicing factor family that participates in the multistep splicing pathway (Graveley 2000). Consequently, these SNPs affect the structure and function of protein as a mutated exon may be skipped by the splicing machinery. Coding SNP rs2274223 in *PLCE1* gene was identified as deleterious to protein coding (Table 3). Moreover, SNP rs2274223 (H1619R) was characterized as intolerant (index, 0.01) and damaging by the SIFT and PolyPhen programs as well (Table 4).

**Table 3:** Functional effect of nsSNPs of *PLCE1* gene predicted by F-SNP and PANTHER

SNPs	Function	FS score	Functional category		PANTHER prediction		
			Protein coding	Splicing regulation	Sub PSEC	P deleterious	P substituted
rs3765524	Missense	0.538	Benign	Changed (ESE, ESR)	−1.89949	0.24964	0.31757
rs2274223	Missense	0.308	Deleterious	Changed (ESE, ESR)	−1.55879	0.19136	0.36899

*FS* functional significance score, *ESE* exonic splicing enhancer, *ESR* exonic splicing regulatory elements, *subPSEC* substitution position-specific evolutionary conservation

**Table 4:** Intolerant and deleterious *PLCE1* SNPs predicted by PolyPhen and VarioWatch

SNPs	Function	AAS	SIFT			PolyPhen	
			Tolerance index	PSIC	Prediction	Risk level	Risk type
rs2274223	Missense	H1619R	0.01	0.999	Probably damaging	High	Protein domain abolished
rs3765524	Missense	T1469I	1.00	1.00	Benign	Low	Conservative change

*AAS* amino acid substitution, *PSIC* position-specific independent counts

### Risk identification

Missense coding SNPs may affect the physicochemical properties of proteins. Missense mutation rs2274223 (H1619R) of *PLCE1* subjected to VarioWatch analysis was associated with high risk to protein structure, and it may affect the protein stability by abolishing the specific domain (Table 4). SNPeffect also predicted that this mutation increases protein aggregation and amylogenic tendency, while rs3765524 may only change chaperone binding (Table 5). The results for each missense SNP obtained by F-SNP, SIFT, PolyPhen, VarioWatch, and SNPeffect were validated by PANTHER. The subPSEC score of rs2274223 also predicted it as deleterious for encoded proteins (Table 3).

**Table 5:** Phenotypic effects of *PLCE1* SNPs on protein structure predicted by SNPeffect

SNPs	dTANGO	dWALTZ	dLIMBO	Prediction
rs2274223	142	18	0	Increased aggregation and amylogenic tendency
rs3765524	0	1.00	−3	Low risk, partially changed chaperone binding

### Modeling and analysis of mutant structure

**Fig. 3:**
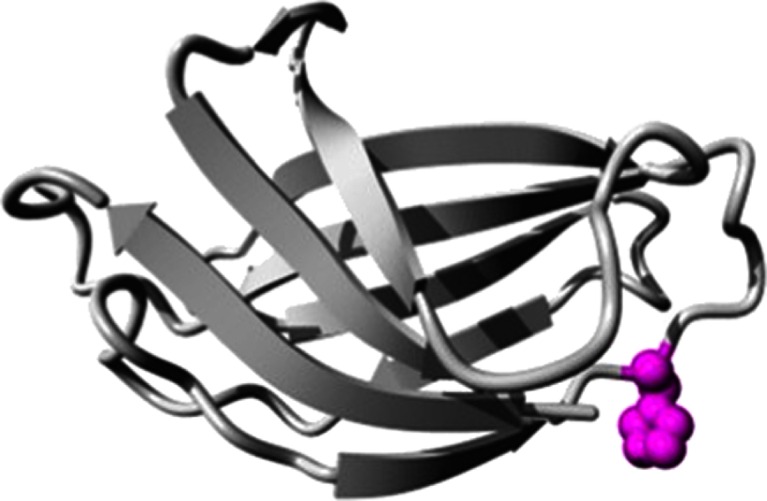
Overview of the predicted protein in ribbon presentation. The side chain mutant residue is shown in *ball shape* and *colored magenta*

**Fig. 4:**
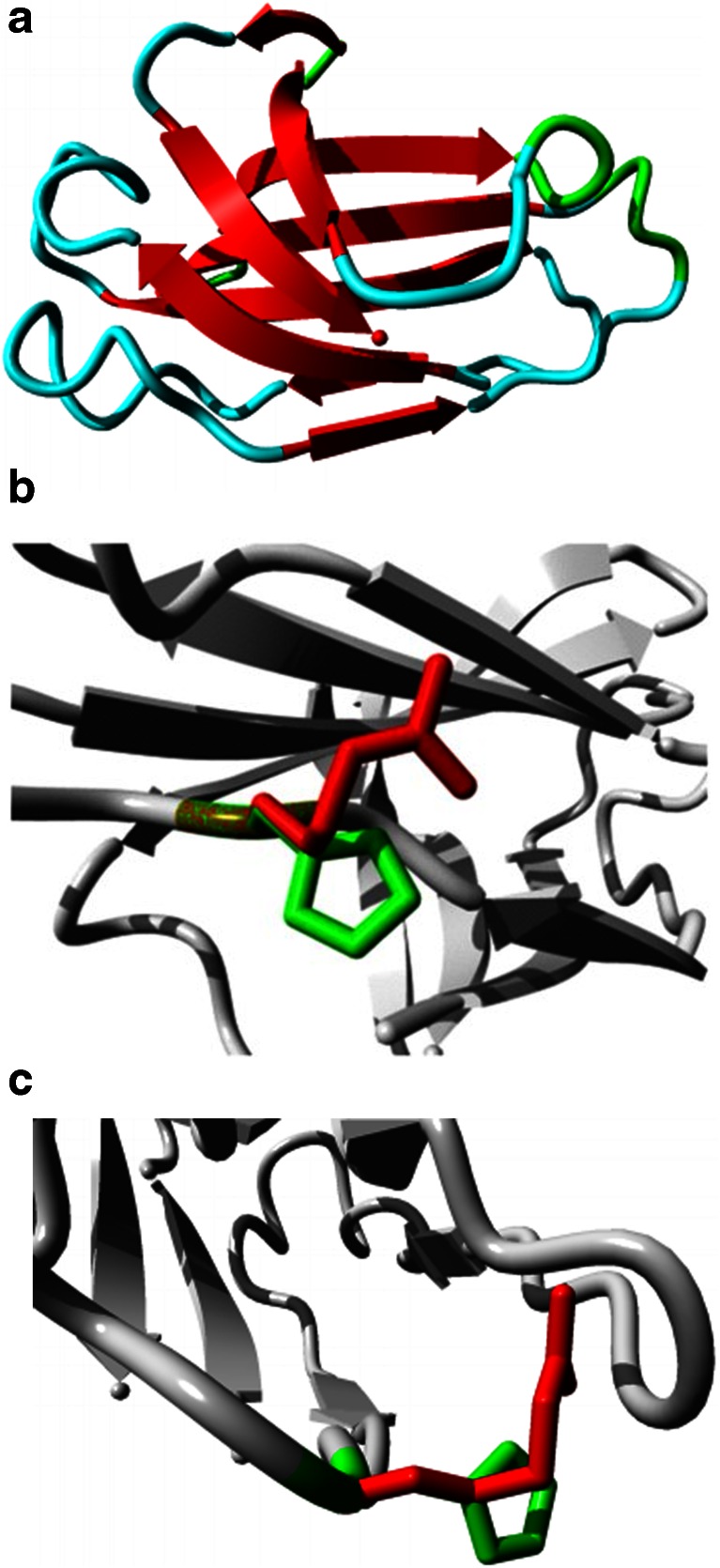
Superimposition of the normal and mutated PLCε proteins. **a** MODELLER-predicted structure of domain (“*C2*_*PLC*_*like*”). Overview of the protein in ribbon presentation. The protein is colored by secondary structure element α-helix in *blue*, β-strands in *red*, turns in *green*, and random coil in *cyan*. This molecular representation is prepared using PyMol. **b** Superimposed structure of protein (“*C2_PLC_like*”) domain. The protein is in *gray* and the wild-type and mutant-type residues are in *green* and *red*, respectively. The protein structure is visualized and prepared in PyMol. **c** Close-up of mutation (seen from a slightly different angle). The protein is in *gray* and the side chains of both the wild type and mutant type are shown in *green* and *red*, respectively

Secondary structural features of the *PLCE1*/*C2* domain present in PLC are illustrated with the mapped mutant residue (Fig. 3). The size of the mutant residue (R) is greater than that of the wild-type residue (H), which might lead to bumps. Moreover, the mutant residue is positively charged and located near a highly conserved position, suggesting probable change in protein structure. This change from neutral (wild type) to positively charged residue may alter protein–protein or protein–ligand interactions. The predicted protein 3D structure is illustrated in Fig. 4a. The wild-type and mutant proteins are superimposed, and the RMSD calculated by superimposing normal and mutated proteins was found to be 1.75 Å (Fig. 4b, c).

The sequence and structure relationship can be better understood with solvent accessibility prediction of residues. NetASA was used to compute the solvent accessibility of all the residues both in the native and the mutant proteins (Shandar Ahmad et al. 2004). It was found that in SNP rs2274223 (H1619R), the histidine residue was buried and not exposed to solvent accessibility, while the mutation of the histidine residue to arginine alters the surface composition of the protein in such a way that the arginine (R) molecule becomes exposed to the surface. In contrast with the SNP (H1619R), the threonine residue of SNP rs3765524 (T1469I) is exposed to the surface in the native protein. Mutation of threonine into isoleucine converts the exposed surface to the buried one as isoleucine was found to be a buried residue via NetASA. Change in solvent accessibility from an exposed to a buried state is considered as functionally significant at the structural level of mutant protein (Chen and Zhou 2005).

To identify the stabilizing residues of the native and mutant structures of protein, SRide server was used (Magyar et al. 2004, 2005). Using the default threshold values (i.e., *E* value for BLAST, 0.001; conservation score threshold, 6; LRO threshold, 0.020; and surrounding hydrophobicity (Hp) threshold, 20.0), we obtained seven stabilizing residue in the native, while five in the mutant structure. This change of stabilizing residues in mutated protein is a clue on the effect of mutation on the stability of protein.

The molecular effect of SNPs was determined by SNPs3D. The wild-type and mutated proteins are compared while considering the wild-type protein as a reference; a set of stability factors (continuous and binary factors) is used to illustrate the impact of each SNP on protein stability (Table 6). The higher the SVM profile score, the more tolerant a mutation is to protein structure. Protein stability is affected by one or more of the following factors: reduction of hydrophobic interactions, loss of hydrogen bonds and salt bridges, overpacking, buried charged and polar residues, electrostatic repulsion, and breakage of disulfide bonds (Wang and Moult 2001).

**Table 6:** Molecular effects of SNP on the 3D structure of protein

Sr. no.	SNP Id	SNP	SVM profile	SVM structure	Molecular effect
1.	rs3765524	T1469I	1.36	0.94	Hydrogen bond lost
2.	rs2274223	H1619R	1.51	1.25	On the protein surface

It was found that in the SNP rs3765524, salt bridge and charged–charged and polar–charged interactions were not lost, while loss of the polar–polar interaction was found to be 0.4 kcal/mol, loss of hydrophobic effect was 0.85 kcal/mol, and one hydrogen bond was lost. While in accordance with the results of the NetASA, the molecular effect of SNP rs2274223 was found, with the hydrophobic effect gained with a value of 0.25 kcal/mol and polar–charged interaction was also gained with 0.05 kcal\mol. This outcome potentially leads toward the alteration of residue from a concealed to an exposed composition, which in turns affects the stability of protein structure.

### Chromatin remodeling

Various histone modifications are caused by *MICB* and *PLCE1* SNPs associated with DSS. It is previously reported that dengue virus-induced enhanced MHC1 expression dampens NK-mediated immune response against dengue virus (Beltrán and López-Vergès 2014). We found that *MICB* SNPs rs3132468 and rs3134899 open the chromatin structure and upregulate the expression of MHC1 protein. On the other hand, *PLCE1* SNPs, dominantly rs2274223 and rs11187842, reduce the expression of PLCε protein by condensing chromatin through inhibitory histone markers. Phospholipase C is an essential signaling effector for T cell proliferation (Table 7). Decline in the activity of PLCε may in turn reduce CD8^+^ cells, leading to enhanced dengue viral load and susceptibility to dengue-triggered complications (Perez-Albuerne et al. 1993). This indicates that histone modification is the key mechanism underlying the association of *PLCE1* and *MICB* SNPs with DSS. It also shows the importance of SNP rs2274223, which impairs not only PLCε structure but also its function.

**Table 7:** Functional effect of *MICB* and *PLCE1* SNPs on histone modifications assessed by the UCSC browser

SNP	Location (hg19)	Histone mark	Cell type	Chromatin
rs3132468	chr6:29778783..32258980	H3k04me1	Hepg2	Open
	chr6:31460736..31485842	H4k20me1	K562	Open
	chr6:31475081..31495127	H3k27me3	K562	Open
	chr6:31456297..31475741	H3k79me2	Dnd41	Open
	chr6:31462215..31479588	H4k20me1	Gm12878	Open
	chr6:31467453..31483972	H3k36me3	Gm12878	Open
	chr6:31471879..31480217	H3k36me3	Dnd41	Open
	chr6:31473210..31479094	H3k36me3	Nhek	Open
	chr6:31474001..31475656	H3k36me3	Hepg2	Open
	chr6:31474699..31476265	H3k36me3	K562	Open
rs3134899	chr6:29778783..32258980	H3k04me1	Hepg2	Open
	chr6:31424359..31477351	H3k9me1	K562	Open
	chr6:31460736..31485842	H4k20me1	K562	Open
	chr6:31462215..31479588	H4k20me1	Gm12878	Open
	chr6:31464188..31474264	H3k79me2	Nhek	Open
	chr6:31467453..31483972	H3k36me3	Gm12878	Open
	chr6:31471879..31480217	H3k36me3	Dnd41	Open
	chr6:31473268..31473768	H3k36me3	K562	Open
rs2274223	chr10:94724854..96159276	H3k27me3	Gm12878	Condense
	chr10:96031821..96090478	H4k20me1	Dnd41	Open
	chr10:96043893..96073132	H3k27me3	K562	Condense
	chr10:96056858..96071667	H3k27me3	Nhek	Condense
	chr10:96065982..96067515	H3k27me3	K562	Condense
	chr10:96066133..96067180	H3k27me3	Gm12878	Condense
rs3781264	chr10:94724854..96159276	H3k27me3	Gm12878	Condense
	chr10:95971943..96291267	H4k20me1	Nhek	Open
	chr10:96031821..96090478	H4k20me1	Dnd41	Open
	chr10:96043893..96073132	H3k27me3	K562	Condense
	chr10:96048848..96075259	H4k20me1	K562	Open
	chr10:96068477..96128278	H3k36me3	Gm12878	Open
	chr10:96056858..96071667	H3k27me3	Nhek	Condense
rs11187842	chr10:94724854..96159276	H3k27me3	Gm12878	Condense
	chr10:96034807..96053564	H3k27me3	Gm12878	Condense
	chr10:96042407..96053597	H3k27me3	Nhek	Condense
	chr10:96043893..96073132	H3k27me3	K562	Condense
	chr10:96052010..96052659	H3k27me3	Pbmc	Condense

## Discussion

Recently, a GWAS reported two susceptible loci associated with DSS—one is MHC class I polypeptide-related sequence B (MICB) and the other is phospholipase C epsilon 1 (PLCE1)—in different populations (Khor et al. 2011; Dang et al. 2014). Although strong evidence of association is provided by these studies, the roles of *MICB* and *PLCE1* genes in the pathogenesis, cross talk of SNPs, and underlying molecular mechanisms for the progression of DSS are not yet fully understood.

A recent study has shown that *MICB* and *PLCE1* genes’ 3′- and 5′-UTR SNPs, which are associated with DSS, are implicated in the worsening of symptoms, from less severe symptoms of dengue to DSS, in children with dengue. Polymorphism in 3′-UTR may regulate transcription efficacy (Barsanjit 2003) The study has shown that the risk allele of *MICB* gene’s UTR SNP was significantly associated with the altered expression of *MICB* gene at the mRNA level (Whitehorn et al. 2013; Dang et al. 2014). *MICB* is associated with NK and CD8 ^+^ cell functions, and it is likely that the presence of SNPs (rs3134899 in this study) reduces the expression of cytokine in NK cells stimulated by *MICB*, resulting in an impaired NK cell response, leading toward higher virus titer and an increased risk of developing DSS (Khor et al. 2011; Libraty et al. 2002). The impaired NK cell response and dysfunctional activity of CD8^+^ cells in dengue-mediated complication is supported by the evidence that SNPs appeared in an allele-specific differential binding affinity for transcription factors (Sakuntabhai et al. 2005). Furthermore, dysregulated T cell responses may also be noticed as a result of inefficient regulatory NK cell induction in clinical phenotypes (Lang et al. 2012).

Moreover, SNP rs753724 in *PLCE1* is involved in regulating and provoking the inflammatory pathways of DSS by recruiting proteins FBJ murine osteosarcoma viral oncogene homolog (Fos) and jun proto-oncogene (Jun) indirectly and forming non-canonical binding motif for SRF directly. Alteration in the binding patterns of the transcription factors of *PLCE1* gene modifies the expression of PLC protein and leads to changes in transcriptional regulation, which in turn regulates the proliferation of CD8^+^ cells (Fu et al. 2010). These SNPs are also involved in chromatin remodeling in such a way that rs3132468 and rs3134899 in *MICB* upregulate the expression of MHC1 protein by opening the chromatin structure, while SNPs in *PLCE1*, rs2274223 and rs11187842, reduce the expression of PLCε protein by condensing chromatin through inhibitory histone markers. The different physiochemical properties and altered binding sites, along with the mutated 3D structure of PLC protein, were observed as a result of missense mutations rs2274223 and rs3765524. SNP rs2274223 alters histidine to arginine in the calcium-dependent lipid-binding (C2) domain of *PLCE1* protein (PLCε). Due to a wide range of lipid selectivity for the cell membrane, the C2 domain is a unique Ca^2+^-dependent membrane-targeting unit involved in calcium-dependent phospholipid binding and signal transduction. Mutation in this domain implies the variation of the conserved region in terms of protein secondary and tertiary structures, which may lead to altered intra- and inter-protein bindings.

Our current data show that the SNPs alter the DNA–protein interaction patterns in an allele-specific manner and lead to the deleterious effects on the encoded proteins’ structure. The current study focused on the mechanistic analysis of *MICB* and *PLCE1* risk genotypes in an Asian population, particularly Vietnamese, so the results should be carefully interpreted for DSS patients from other ethnic backgrounds. Collectively, our findings provide underlying molecular mechanisms of DSS association with *MICB* and *PLCE1* and validate further the importance of *MICB* and *PLCE1* risk genotypes with their roles in disease pathogenesis.

## Conclusion

This study predicts the implication of single nucleotide transition at SNPs in differential protein binding patterns with *PLCE1* and *MICB* genes. It suggests that *PLCE1* SNP rs2274223 has a) deleterious effects on the structure (C2 domain) and function of PLCε protein, b) may implicated in protein aggregation, amylogenic tendency and condensing chromatin which may lead to the pathogenesis of DSS. These findings will enable us to conduct follow-up studies to retrieve novel molecular targets, such as mutated protein domain and modified histone sites, to design effective therapies to either prevent DSS onset or impede its progression.

## Abbreviations

AAS: Amino acid substitution
DSS: Dengue shock syndrome
FS: Functional significance
NK: Natural killer cells
